# Reduction in BDNF from Inefficient Precursor Conversion Influences Nest Building and Promotes Depressive-Like Behavior in Mice

**DOI:** 10.3390/ijms21113984

**Published:** 2020-06-01

**Authors:** Masami Kojima, Hikari Otabi, Haruko Kumanogoh, Atsushi Toyoda, Masahito Ikawa, Masaru Okabe, Toshiyuki Mizui

**Affiliations:** 1 Biomedical Research Institute, National Institute of Advanced Industrial Science and Technology (AIST), 1-8-31 Midorioka, Ikeda, Osaka 563-8577, Japan; h.kumanogoh@gmail.com; 2 Core Research for Evolutional Science and Technology (CREST), Kawaguchi 332-0012, Japan; 3 Graduate School of Frontier Bioscience, Osaka University, Suita 565-0871, Japan; 4 College of Agriculture, Ibaraki University, Ami, Ibaraki 300-0393, Japan; s175911s@st.go.tuat.ac.jp (H.O.); atsushi.toyoda.0516@vc.ibaraki.ac.jp (A.T.); 5 Research Institute for Microbial Diseases, Osaka University, Suita, Osaka 5650871, Japan; ikawa@biken.osaka-u.ac.jp (M.I.); okabe@biken.osaka-u.ac.jp (M.O.)

**Keywords:** BDNF, depression, nest building, cleavage, SNP, social isolation

## Abstract

We generated a knock-in mouse line in which the gene encoding brain-derived neurotrophic factor (*Bdnf*) was replaced with a sequence for proBDNF containing human single nucleotide polymorphisms encoding arginines proximal to the cleavage site (R125M and R127L). The ratio of the mature form of BDNF (mBDNF) to precursor BDNF (proBDNF) in hippocampal tissue lysates was decreased in a manner dependent on the number of copies of the mutant gene, indicating that the mutations inhibited proteolytic conversion of proBDNF into mBDNF. Although homozygous mice had a proBDNF/mBDNF ratio of ~9:1, they survived until adulthood. The levels of mBDNF were reduced by 57% in heterozygous mutant mice, which exhibited a depressive-like behavior in the tail suspension test and weight gain when housed in social isolation, showing that impaired proBDNF cleavage contributes to stress-induced depressive-like phenotypes. Furthermore, socially isolated heterozygous mice displayed a pronounced deficit in daily nest-building behaviors. These findings suggest that the decreased production of mBDNF by impaired proBDNF cleavage disturbs daily activities in mice.

## 1. Introduction

Brain-derived neurotrophic factor (BDNF; mBDNF—mature form of BDNF) promotes the survival of developing peripheral neurons [1,2] and has trophic effects on a variety of neuronal types [3]. mBDNF is highly expressed in many brain regions in response to neuronal activity [4,5] and facilitates activity-dependent synaptic plasticity via tropomyosin-related kinase B (TrkB) receptors [6]. Brain function and activity-dependent secretion of mBDNF are disrupted in individuals expressing a common variant of mBDNF with a single nucleotide polymorphism (SNP) (reference SNP cluster ID rs6265), resulting in the replacement of the valine at residue 66 in the pro-peptide region with methionine (Val66Met) [7]. Recent reports suggest that impaired mBDNF signaling contributes to depression and that antidepressants can restore mBDNF expression [8,9,10]. Moreover, postmortem brain samples from depressed patients have reduced amounts of mBDNF and of the TrkB receptor [11,12,13,14], and TrkB phosphorylation is reduced in suicide victims [15].

Various rodent models have been used to explore the role of mBDNF in depression. Male mice with reduced levels of BDNF in forebrain regions exhibit depressive-like behavior [16], and knockdown of BDNF in the dentate gyrus area of the hippocampus in rats produces depressive-like effects [17]. However, depressive-like behaviors were not reported for mice overexpressing a nonfunctional TrkB receptor in the forebrain [18,19] or for mice lacking one *Bdnf* allele (BDNF^+/−^ mice) [18,20,21], although these mice were slower to escape in the learned helplessness paradigm of depression [20]. It is also indicated that depressive-like behaviors are induced by exposure to stress, which results in reduced levels of mBDNF in the cortex and hippocampus in animal models [22,23,24]. In addition, Mishima et al. reported that mice deficient for Ca^2+^-dependent activator protein for secretion 2 (CAPS2), a regulator of dense-core vesicle exocytosis that is involved in the secretion of mBDNF and NT-3, display depressive-like behaviors after chronic treatment with corticosterone [25].

BDNF transcription through its multiple promoters, its trafficking, and its secretion have all been implicated in the onset and development of depression [26]. mBDNF, like other neurotrophins, is synthetized as a precursor protein (proBDNF; molecular mass of ~32 kDa) that is proteolytically processed into mature BDNF (mBDNF; molecular mass of ~14 kDa) by intracellular and/or extracellular proteases, most prominently by the proprotein convertase PC7, but also extracellularly by metalloproteinases and plasmin (reviewed by Lessmann [4]). Several reports demonstrate that proBDNF promotes cell death, growth cone retraction, dendritic spine shrinkage, and long-term depression, whereas mBDNF promotes neuronal survival, spine protrusion, and long-term potentiation [27,28,29].

**Figure 1:**
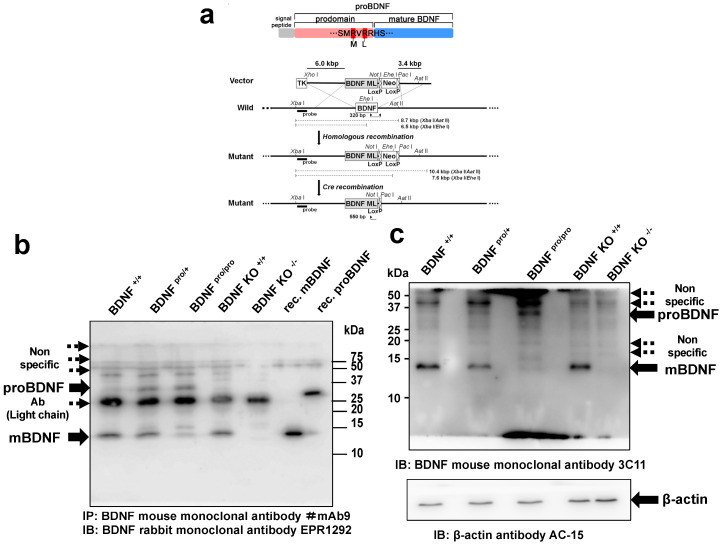
Generation of a knock-in mouse line expressing low levels of mature brain-derived neurotrophic factor (mBDNF). (**a**) Strategy used to replace the coding region of *Bdnf* with cDNA encoding cleavage-resistant proBDNF. (Top) The cleavage-resistant form of BDNF contains two amino acid substitutions proximal to the cleavage site of proBDNF. (Bottom) The wild-type allele yields an 8.7-kb fragment, and the mutant allele yields a 10.4-kb fragment with XbaI/AatII digestion of genomic DNA. XbaI/EheI digestion results in a 6.5-kb fragment in the wild type and a 7.6-kb fragment in the mutant. Broken lines in the upper schema indicate the approximate locations of these four fragments. (**b**) Western blot showing the influence of amino acid substitutions (R→M, R→L in panel a) proximal to the cleavage site of proBDNF on the expression of proBDNF and mature BDNF. Hippocampal lysates (containing 500 µg protein) were incubated with a mouse monoclonal antibody (clone; mAb#9) recognizing both proBDNF and mature BDNF [32], and Western blotting was performed with a rabbit anti-pan-BDNF monoclonal antibody (clone; EPR1292; Abcam); BDNF^+/+^, BDNF^pro/+^, BDNF^pro/pro^ mice were 8 weeks old, and BDNF KO^+/+^ BDNF KO^−/−^ mice were 2 weeks old. (**c**) Immunoblot analysis of mBDNF and proBDNF in hippocampal lysate (25μg protein/lane). BDNF^+/+^, BDNF^pro/+^, BDNF^pro/pro^ mice were 8 weeks old, and BDNF KO^+/+^ BDNF KO^−/−^ mice were 9 days old. Note that mBDNF and proBDNF were detected with a mouse anti-pan-BDNF monoclonal antibody (clone; 3C11, Icosagen), followed by Wosnitzka et al. (2020) [34]. Membranes have been re-probed with a mouse anti-β-actin monoclonal antibody (clone; AC15, Sigma) to check for an equal amount of protein.

In addition to a common BDNF polymorphism, V66M (rs6265) [7], we explored the molecular roles of rare tandem SNPs at nucleotides 373 (G/T) and 379 (G/T) (rs1048220 and rs1048221, respectively) in the coding region of the human BDNF gene [29]. Notably, these SNPs are located near the cleavage site of proBDNF (Figure 1a) and code for a methionine in place of arginine at position 125 (R125M) and leucine at position 127 (R127L), respectively (Figure 1a). In our previous report, a bioinformatic analysis predicted that these variations undergo disordered-to-ordered transitions around the cleavage site of proBDNF [29]. In in vitro studies, the proteolytic conversion of the mutant proBDNF construct into mBDNF was impaired and induced shrinkage of dendritic spines in cultured hippocampal neurons [29]. As a reduction in mBDNF is associated with depression, as described above, impairment of proBDNF cleavage may represent a mechanism by which these and similar SNPs influence the development of psychiatric phenotypes in mice.

In the present study, we generated a knock-in mouse line in which the endogenous *Bdnf* allele was replaced with cDNA encoding human BDNF, in which two arginine residues located proximal to the cleavage site are mutated (Figure 1a). These mutations markedly impaired the conversion of proBDNF into mBDNF, demonstrating that these arginine residues are crucial for proBDNF cleavage in vivo. Heterozygous knock-in mice, which produce approximately 50% less mBDNF than wild-type littermates, exhibited a pronounced depressive-like behavior and impaired nest building when housed for eight weeks under conditions of social isolation. These findings suggest that proteolytic cleavage of proBDNF plays pathophysiological roles in the daily activities of mice and the susceptibility to mood disorders, such as depression-like behaviors.

## 2. Results

### 2.1. Generation of a Knock-in Mouse Line with Impaired Conversion of proBDNF into mBDNF

We generated a knock-in mouse line using the homologous recombination strategy described previously [30]. Specifically, the endogenous *Bdnf* allele was replaced with a sequence encoding proBDNF containing rare human tandem SNPs, at nucleotides 373 (G/T) and 379 (G/T), which change two arginines (amino acids 125 and 127) proximal to the cleavage site to methionine and leucine, respectively, near the cleavage site of proBDNF (Figure 1a, top). We previously reported that these mutations result in inefficient conversion of proBDNF to mBDNF in cultured neurons [29]. In the present article, homozygous and heterozygous knock-in mice are referred to as BDNF^pro/pro^ and BDNF^pro/+^, respectively.

BDNF proteins are highly expressed in hippocampus [31], and Rauskolb et al. (2010) [32] determined the ratio of mBDNF to proBDNF in hippocampal tissues of 9-week-old mice by immunoprecipitation followed by Western blotting (IP-WB). We used this method to first analyze how efficiently proBDNF was converted into mBDNF in the hippocampi of 9-week-old BDNF^+/+^, BDNF^+/pro^, and BDNF^pro/pro^ littermate mice. We used a mouse monoclonal antibody (clone; mAb#9) recognizing both proBDNF and mature BDNF for IP [32] and a mouse anti-pan-BDNF antibody (clone; EPR1292; Abcam) for the WB. Consistent with the findings of Rauskolb et al. (2010) [32], mBDNF expression was much higher than that of proBDNF in control mice (Figure 1b, BDNF^+/+^). Notably, the ratio of mBDNF to proBDNF appears to decrease in a manner dependent on the number of copies of the mutant gene, indicating the amino acid substitution of the first and second arginines proximal to the cleavage site to methionine and leucine (see Figure 1a, top; R→ M and R→ L) inhibits proteolytic conversion of proBDNF into mBDNF in vivo, as was observed in vitro [29]. Although nonspecific bands with higher and lower molecular masses were also seen, the findings were confirmed by including positive and negative controls with hippocampal lysates from 10-day-old wild-type (BDNF KO^+/+^) and BDNF-null (BDNF KO^−/−^) mice. Neither mBDNF nor proBDNF bands were detected in BDNF KO^−/−^ lysates (Figure 1b, BDNF KO^−/−^). While mBDNF was abundant in the lysates of BDNF KO^+/+^ mice (Figure 1b, BDNF KO^+/+^, mBDNF), proBDNF was not detectable, probably because the BDNF levels are not high in the developing mouse [32]. Furthermore, we loaded recombinant (rec.) mBDNF and proBDNF generated in *E. coli* (Figure 1b). As the *E. coli*-derived proBDNF is not glycosylated [33], the corresponding band had a lower molecular mass than that for proBDNF in mouse lysates. On the basis of these findings, we reason that the two rare human SNPs proximal to the cleavage site of proBDNF cause inefficient cleavage, resulting in decreased levels of mBDNF in the hippocampus in vivo in a manner dependent on mutant gene dosage.

Further, to investigate the ratio of mBDNF and proBDNF, we performed Western blot analysis of the hippocampal lysates prepared from 9-week-old BDNF^+/+^, BDNF^+/pro^, and BDNF^pro/pro^ littermate mice. To detect the immunoreactive signals of mBDNF and proBDNF, we used a monoclonal anti-pan-BDNF antibody (clone; 3C11, Icosagen) as reported by Wosnitzka et al. (2020) because they showed that mBDNF and proBDNF were detected in Western blot analysis using antibody 3C11 in HEK293 cell lysate transfected with BDNF or BDNF-GFP fusion, while their respective signals were not seen in a Western blot of the lysates prepared from the cerebral cortex of Bdnf−/− animals [34]. In line with the result of Figure 1b, the ratio of mBDNF to proBDNF decreased in a manner dependent on the number of copies of the mutant gene (Figure 1c, arrows). The bands corresponding to mBDNF and proBDNF were not clearly seen in the lanes loaded with the lysates prepared from the cerebral cortex of 9-day-old BDNF KO^−/−^ animals (Figure 1c, BDNF KO^−/−^), indicating that the 3C11 antibody is available for Western blotting of mBDNF and proBDNF. Furthermore, a quantitative analysis indicated that the percentage of mBDNF to proBDNF in BDNF^+/+^, BDNF^pro/+^, and BDNF^pro/pro^ hippocampal tissues were 92.1 ± 2.8%, 57.7% ± 10.5%, and 6.3% ± 2.5%, respectively (*n* = 3 independent mice). 

### 2.2. Body Weight and Heart Morphology

**Figure 2:**
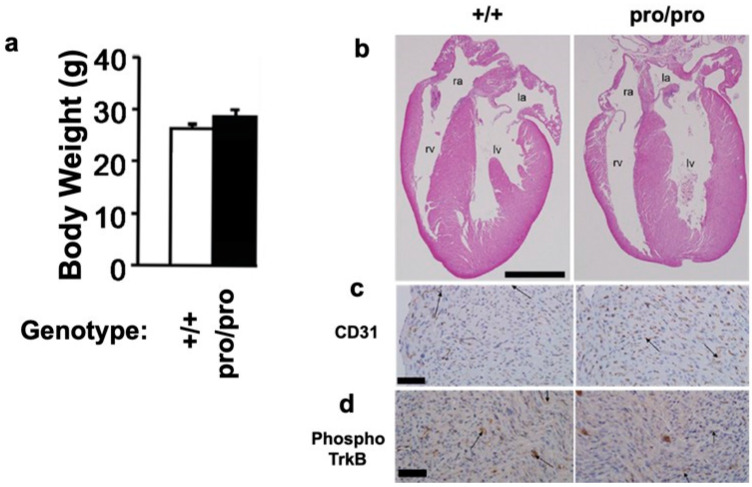
Body weights and heart morphology. (**a**) Mean body weights of 10-week-old BDNF^+/+^ and BDNF^pro/pro^ animals. Error bars represent SEMs from 8 mice/group. (**b**) Hematoxylin and eosin-stained heart sections of 7-day-old mice with the indicated genotypes. The staining was performed as described by Donovan et al. [35]. Abbreviations: ra, right atrium; la, left atrium; rv, right ventricle; lv, left ventricle. (**c**,**d**) Immunostaining of BDNF^+/+^ and BDNF^pro/pro^ heart sections using an antibody against CD31 (**c**) or phosphorylated TrkB. (**d**) Scale bars, 1 mm (**b**) and 50 mm (**c**,**d**).

The proBDNF knock-in gene followed a Mendelian inheritance pattern (Table S1). The homozygous mutant mice survived to adulthood, and the body weights of BDNF^+/+^ and BDNF^pro/pro^ littermate mice were comparable (Figure 2a). These results were somewhat unexpected, because *Bdnf* null mice die as a result of severe body size reduction, heart malformation, and intramyocardial hemorrhage [35]. However, 7-day-old BDNF^pro/pro^ mice did not exhibit heart malformations or intramyocardial hemorrhages (Figure 2b). An immunohistochemical analysis of endothelial marker CD31 indicated that the endothelial cells in the hearts of BDNF^pro/pro^ mice were morphologically normal (Figure 2c). Similarly, immunostaining for phosphorylated TrkB indicated normal activation of this receptor (Figure 2d). Together, these results suggest that inefficient cleavage of proBDNF to mBDNF does not influence animal survival or heart morphology.

### 2.3. Impact of Social Isolation on Depressive-Like Behavior of BDNF^pro/+^ Mice

**Figure 3:**
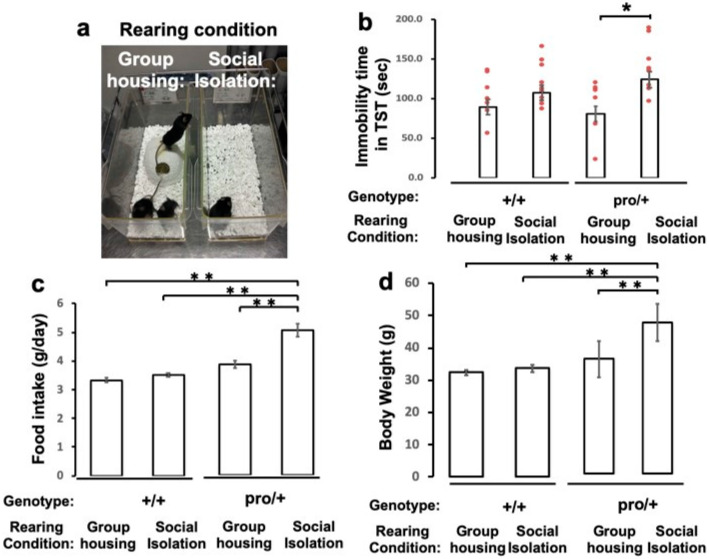
Behavioral phenotypes of BDNF^pro/+^ mice housed for 8 weeks in social isolation. (**a**) Housing conditions. Three mice were housed in each cage for group housing, whereas one mouse was housed in each social isolation cage (according to Wallace et al. [36] with some modification). (**b**) After 8 weeks, mice housed in group or social isolation cages were subjected to the tail suspension test, as described in Materials and Methods. (**c**) Food intake was measured every day for 8 weeks, and the mean daily amount of food intake was determined. (**d**) Body weights were measured after the tail suspension test. Data are presented as means ± SEMs from 10–12 animals/group. * *p* < 0.05, ** *p* < 0.001, one-way ANOVA followed by Tukey–Kramer test.

To study if the conversion of proBDNF into mBDNF is associated with the development of depressive-like behaviors, we reared 6-week-old male BDNF^pro/+^ heterozygous mice, in which mBDNF levels were reduced to 57.7% ± 10.5% of that in BDNF^+/+^ mice (*n* = 3 independent mice), and their age-matched wild-type littermates under conditions of social isolation or group housing for 8 weeks (Figure 3a) according to a previous report [36]. To assess depressive-like behaviors, we measured immobility times in the tail suspension test, which is thought to measure despair under extreme adversity [37]. The BDNF^pro/+^ mice reared in social isolation exhibited approximately 2-fold longer immobility times than group-housed BDNF^pro/+^ animals (Figure 3b; *p* < 0.05, ANOVA followed by Tukey–Kramer test). By contrast, there was no difference in immobility between BDNF^+/+^ mice housed in groups or in social isolation (Figure 3b). Furthermore, BDNF^pro/+^ mice housed in social isolation for 8 weeks showed significantly increased food intake and body weights compared to those housed in groups. Social isolation did not induce increases in weight or food intake in wild-type mice (Figure 3c,d; *p* < 0.001, one-way ANOVA followed by Tukey–Kramer test). These results suggest that impaired cleavage of proBDNF into mBDNF promotes depressive-like behaviors, observed as increased mobility in the tail suspension test and increased food intake and body weight in socially isolated BDNF^pro/+^ mice.

### 2.4. Impaired Nest Building in Socially Isolated BDNF^pro/+^ Mice

**Figure 4:**
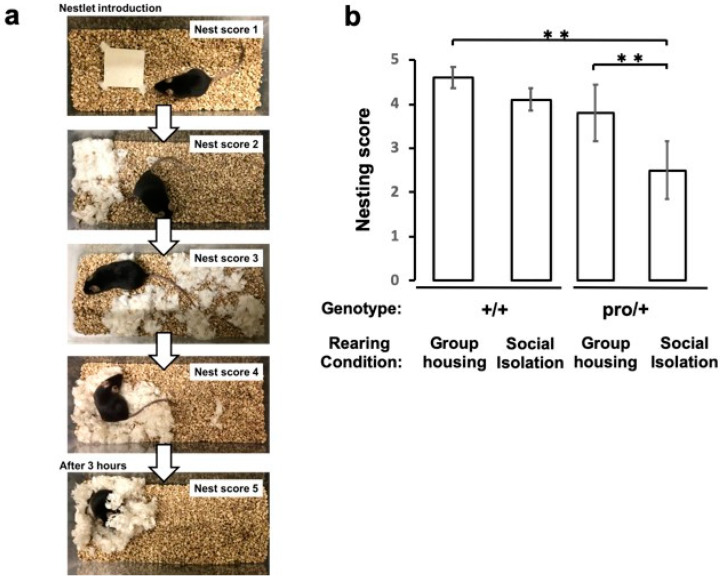
Deficit in nest building by socially isolated BDNF^pro/+^ mice. (**a**) Nest-building scoring (from 1 to 5, as described in Materials and Methods). (**b**) BDNF^pro/+^ and BDNF^+/+^ mice were housed in group or social isolation cages for 8 weeks before they were subjected to the nest-building behavioral test. Data are presented as the means ± SEMs from ten animals/group (** *p* < 0.001, one-way ANOVA followed by Tukey–Kramer test).

The construction of a nest is a deliberate behavior performed by mice to improve fitness and survival. To see if altered BDNF levels influence this behavior, we first housed BDNF^pro/+^ and BDNF^+/+^ mice for 8 weeks under social isolation or group-housed conditions and then scored their 12 h nest-building behavior according to the protocol described by Deacon [38,39]. BDNF^+/+^ mice showed interest in nest materials, shredding the materials and completing their nests within 3–4 h (Figure 4a). However, socially isolated BDNF^pro/+^ mice exhibited significantly poorer nest building than group-housed BDNF^+/+^ and BDNF^pro/+^ mice (Figure 4b; *p* < 0.001, one-way ANOVA followed by Tukey–Kramer test). These results suggest that deficient proBDNF cleavage influences a normal survival behavior.

## 3. Discussion

The in vivo roles of mBDNF and related molecules in depression have been extensively explored in genetically engineered mouse lines [40]. To investigate this in a more pathophysiologically relevant context, we focused on *Bdnf* alleles with a sequence containing two rare human SNPs proximal to the cleavage site of proBDNF. A bioinformatic analysis performed previously [29] indicated that these SNPs would interrupt proteolytic cleavage. In the present study, we generated knock-in mice in which the gene encoding *Bdnf* was replaced with a sequence for proBDNF containing human single nucleotide polymorphisms encoding arginines proximal to the cleavage site (R125M and R127L) and demonstrated that these mutations in the *Bdnf* gene lead to a gene dosage-dependent decrease in the levels of mBDNF as a result of inefficient cleavage of proBDNF.

Second, we showed that the homozygous mice survived until adulthood, unlike *Bdnf* null mice that die at an early postnatal age as a result of severe heart malformation and intramyocardial hemorrhage [35]. However, no such defects were observed in BDNF^pro/pro^ mice. This may be explained by the residual levels of mBDNF observed in our homozygous mice. Yang et al. generated a knock-in mouse line with mutations at the second and third arginines (rather than the first and second), which completely inhibited proBDNF cleavage [41]. These two studies using knock-in mice with distinct mutations suggest that the three arginine residues located near the cleavage site of proBDNF play different roles in the proteolytic cleavage of proBDNF.

To verify that mutations (R→ M, R→ L, Figure 1a) impair proBDNF cleavage, we examined the expression of mBDNF and proBDNF. As BDNF proteins were not abundant in the hippocampal lysates, IP-WB was performed according to the method described by Rauskolb et al. (2010) [32]. The experiments showed that mBDNF is expressed at a higher level than proBDNF wild-type animals (Figure 1b), in line with the notion that proBDNF is rapidly converted into mBDNF mainly in naïve neurons [42]. Further, we confirmed the presence of proBDNF in wild-type mice via immunoprecipitation with a mouse monoclonal antibody recognizing the BDNF pro-domain (3C10H), which recognizes proBDNF but not mBDNF, followed by Western blotting with a rabbit anti-pan-BDNF monoclonal antibody (EPR1292) (Figure S1, BDNF KO^+/+^); the lysates prepared from BDNF KO^-/-^ mice did not contain proBDNF. Furthermore, Western blot analysis was performed to examine the ratio of mBDNF to proBDNF using a monoclonal anti-BDNF antibody (clone; 3C11, Icosagen). Until now, the confusing results of biochemistry would have arisen from the use of unsuitable antibodies. However, Wosnitzka et al. (2020) showed that BDNF antibody 3C11 detected both mBDNF and pro-BDNF with transfected cells and that these bands were not seen in Western blot when used with lysates prepared from Bdnf^−/−^ animals [34]. Using the mouse anti-pan-BDNF monoclonal antibody 3C11, percentage of mBDNF to proBDNF in BDNF^+/+^, BDNF^pro/+^, and BDNF^pro/pro^ in mice was 92.1 ± 2.8%, 57.7% ± 10.5%, and 6.3% ± 2.5%, respectively. These findings indicate that the ratio of mBDNF to proBDNF depends on the number of copies of the mutant gene.

The neurotrophic hypothesis of depression states that a deficiency in neurotrophic support contributes to the development of depression, and most related studies have focused on the neurotrophin mBDNF [40]. However, reductions in mBDNF levels or TrkB signaling are not solely responsible for depressive-like behaviors, because mice lacking one allele of *Bdnf* resulting in a 50% reduction in mBDNF levels do not exhibit depressive-related behaviors, whereas female mice with specific loss of mBDNF expression in forebrain exhibited depressive-like behaviors [16]. Given that another study showed that knockdown of BDNF in the dentate gyrus induces depression-like effects in rats [17], the role of BDNF in depression needs further investigation at molecular cellular levels. 

In the present study, we demonstrated that mBDNF levels in the hippocampus are reduced by approximately 57% in BDNF^pro/+^ heterozygous mice and that these mice exhibit depressive-like behaviors after being housed in social isolation. As BDNF^pro/+^ mutant mice exhibited reduced mBDNF levels as a result of inefficient cleavage of proBDNF and spent more time immobile in the tail suspension test after being housed in social isolation, the stress of social isolation and impaired proBDNF cleavage may cooperatively promote depressive-like behavior. In line with these findings, it was reported that the ratio of mBDNF to proBDNF increases in mice subjected to chronic unpredicted mild stress that exhibit depressive-like behaviors [43] as well as in a rat model of anxiety induced by intraplantar injections of complete Freund’s adjuvant and a model of depression induced by chronic restraint stress [44]. Moreover, a clinical report indicated that the mBDNF/proBDNF ratio in serum is a potential biomarker to discriminate patients with bipolar depression among those with depressive episodes [45]. 

Stress, a risk factor for depression [46], influences the function of mBDNF and synapse in brain [47,48]. It was demonstrated that mice exposed to social stress exhibited BDNF downregulation [22]. Notably, Hajszan et al. performed quantitative electron microscopic stereology and demonstrated that depression, stress, and corticosterone administration lead to hippocampal spine synapse loss and shrinkage of pyramidal cell dendritic trees [49]. Our in vitro study showed that the impairment of proBDNF cleavage resulted in the shrinkage of the morphology of hippocampal dendritic spines in culture [29]. Thus, it would be interesting to examine in the future whether proBDNF cleavage, a posttranslational mechanism of mBDNF production, contributes to such remodeling of spine synapses with stress and depression.

Most research of depression has focused on the hippocampus and frontal cortex. However, these limbic regions interconnect with subcortical structures that contribute to various symptoms of depression [26], such as food intake and body weight. Indeed, mBDNF is highly expressed in the hypothalamus as well as the hippocampus [31]. Recently, it was demonstrated that signaling through TrkB in the hypothalamus was crucial for controlling satiety and body weight (reviewed by Xu and Xie (2016) [50]). In the present study, BDNF^pro/+^ mice housed in social isolation increased their food intake and body weights. Thus, taken together, singly housed BDNF^pro/+^ mice, which exhibit increased food intake and body weights, may have impaired hypothalamic mBDNF signaling. 

Interestingly, it was previously reported that *Bdnf* gene expression in the hypothalamus induced by environmental enrichment induces the browning of white adipocytes [51]. However, *Bdnf* expression was not different between BDNF^pro/+^ and BDNF^+/+^ mice housed in social isolation (Figure S2). We further found that the total amount of BDNF-immunoreactive protein (Mbdnf + proBDNF) was not different between the genotypes (1 ± 0.07 [BDNF^+/+^] versus 1.16 ± 0.10 [BDNF^pro/+^]; *p* = 0.30 by *t* test, *n* = 3 mice). Therefore, since the percentage of mBDNF to proBDNF was lower in BDNF^pro/+^ mice than that of BDNF^+/+^ animals (Figure 1b,c), the observed increases in weight or food intake in BDNF^pro/+^ animals might be attributable to the reduction of mBDNF levels. However, in hypothalamus, BDNF expresses in some neural circuits [50]. To understand the pathophysiological influence of impaired processing of proBDNF in the hypothalamic function, we will study the ratio of mBDNF to proBDNF using the tissues including BDNF-expressing neural circuits in BDNF^pro/+^ hypothalamus in the future.

A surprising finding in the present study is that BDNF^pro/+^ mice housed alone show an impairment in nest-building behavior. These data suggest that mBDNF plays an important role in this natural daily behavior. Nest building is an innate but complex goal-directed behavior [52,53] that requires neural processes in the frontal cortex and hippocampus [54,55,56,57,58], two brain regions that express mBDNF [31]. Thus, reduced levels of mBDNF may impair complex goal-directed and natural daily behaviors. Nevertheless, we cannot rule out the contribution of decreased concentration on task completion or impaired motivation, both of which are cardinal signs of major depressive disorder, because the BDNF^pro/+^ mice housed alone exhibited depressive-like behaviors. We recently reported a pronounced impairment of nesting behavior in socially defeated mice [59]. This nesting deficit was partially rescued by the serotonin 2A (5-HT2A) receptor antagonist [60], which was also found to block the stress-induced downregulation of *Bdnf* mRNA expression in hippocampus [61]. Furthermore, an increase in the 5-HT2A receptor in the hippocampus has also been observed in depressed patients [62]. Given these findings, mBDNF and serotonin signaling might contribute to the performance of daily behaviors, which should be explored in more detail in the future. 

## 4. Materials and Methods

### 4.1. Animals, Breeding Procedures, Housing Conditions, and Animal Ages at Each Experiment

All animal experiments were performed in strict accordance with protocols that were approved by the Institutional Animal Care and Use Committee of AIST. All efforts were made to minimize animal suffering during the experiments (number.2019-084, approval date 6 June 2015). Wild-type mice with the background of C57BL/6J were obtained from Crea Japan (Shizuoka, Japan). Mice were housed with access to regular laboratory chow (MF; Oriental Mouse Food, Tokyo, Japan) and water ad libitum at constant room temperature (24 ± 2 °C) and were exposed to a 12 h light/dark cycle (lights on from 7 a.m. to 7 p.m.). All mice were housed in groups of three per cage prior to the experiments. Social isolation paradigm is according to the report by Wallace et al. [36] with some modification. For the 8 weeks of social isolation, mice were moved to individual cages at 6 weeks of age. The approximate ages of the mice when each experiment was performed are as follows: immunoblotting and immunocytochemistry, 8 weeks old; measurement of body weight, 10 weeks old; hematoxylin and eosin staining, 7 days old; behavioral tests, 14 weeks old.

### 4.2. Generation of the proBDNF Knock-in Mouse Line

The proBDNF knock-in mouse line was generated by replacing the endogenous *Bdnf* allele with cDNA encoding two mutations proximal to the cleavage site of proBDNF (RVRR to MVLR), resulting in a cleavage-resistant form of BDNF using the methods described by Inoue et al. [30]. Briefly, a 6.0-kb long-arm fragment (NCBI accession number: AY057907, 49015–54460) and a 3.4-kb short-arm fragment (55207–58754) flanking the 5′ and 3′ ends, respectively, of mouse *Bdnf* exon 5 were PCR amplified from 129SV mouse genomic DNA. These fragments were introduced into the ClaI-NotI and PacI-AscI sites, respectively, of the pMulti-ND 1.0 vector. A DNA fragment encoding mutant proBDNF was then inserted into the ClaI-NotI site of the targeting vector. A pGK-thymidine kinase gene was used as a negative selectable marker in the vector (see Figure 1a). The linearized targeting vector was electroporated into embryonic stem (ES) cells of the D3 line (strain 129SV). DNA derived from G418-resistant ES clones was screened by PCR, and positive ES clones were injected into blastocysts obtained from C57BL/6J mice. The injected blastocysts were then introduced into the uteri of pseudopregnant females. Chimeric mice were mated with C57BL/6J mice to produce heterozygotes, and these mice were subsequently crossed with mice expressing Cre recombinase in germ cells to excise the neo cassette [30]. Genetic backcrosses to the C57BL/6J and 129SV backgrounds were performed for at least ten generations before the animals were used in behavioral analyses and in all other analyses, respectively. Thus, proBDNF knock-in mice have a C57BL/6J genetic background.

### 4.3. Genotyping

Genotyping of the proBDNF knock-in mouse line was performed using the following primers: 5′-TGCACCACCAACTGCTTAG-3′ and 5′-GGATGCAGGGATGATGTTC-3′. PCR analyses using these primers generated 550-bp and 320-bp DNA fragments from wild-type and mutant alleles, respectively. Genotyping of the BDNF knock-out mice was performed using the following primers: 5′-ATGAAAGAAGTAAACGTCCAC-3′, 5′-CCAGCAGAAAGAGTAGAGGAG-3′, and 5′-GGGAACTTCCTGACTAGGGG-3′. PCR analyses using these primers generated 275-bp and 340-bp DNA fragments from wild-type and mutant alleles, respectively.

### 4.4. Generation of Anti-proBDNF Antibody

A peptide with the sequence KVRPNEENNKDADLY (aa 76–90) [33] was synthesized via a fluorenyl methoxy carbonyl-based liquid-phase peptide synthesis process. The peptide was conjugated with keyhole limpet hemocyanin, emulsified with Freund’s adjuvant (Pierce, Rockford, IL, USA), and then used to immunize female mice to screen hybridoma clones. Clone 3C10H was selected from the initial screening and further characterized using hippocampal lysates from BDNF KO and wild-type littermates as shown in Figure S1.

### 4.5. Immunoprecipitation and Immunoblot Analysis

A purified mouse monoclonal antibody recognizing BDNF and proBDNF (clone; mAb#9) was generously provided by Professor Yves-Alain Barde (School of Biosciences, Cardiff University, United Kingdom). First, hippocampi were dissected from 9- or 2-week-old mice of the designated genotypes, weighed, and stored at −80 °C. The dissected hippocampi were lysed in RIPA buffer (Thermo Scientific, Waltham, MA, USA) containing a protease inhibitor mixture (Roche Diagnostics, Basel, Switzerland) using a sonicator (TAITEC, Saitama, Japan), and the supernatants of the lysates were collected after centrifugation at 20,400× *g* for 10 min. The protein concentration of the supernatants was determined using a BCA assay kit (Thermo Scientific). The supernatants containing 500 µg of total protein were incubated with the BDNF monoclonal antibody (mAb#9) or proBDNF monoclonal antibody (3C10H) at 4 °C for 24 h and subsequently incubated with protein G Sepharose beads (GE Healthcare Life Sciences, Piscataway, NJ, USA) at 4 °C for 2 h. Precipitates were collected by centrifugation, eluted with sample buffer, and stored at −80 °C.

The precipitates were boiled for 5 min at 100 °C, electrophoresed on 20% SDS-polyacrylamide gels, and then transferred to polyvinylidene fluoride membranes using the Trans-Blot Turbo transfer system (Bio-Rad, Hercules, CA, USA). The membranes were blocked in Tris-buffered saline containing 0.2% Tween-20 (TBST) and 5% non-fat dry milk (WAKO, Osaka, Japan), incubated with a rabbit anti-pan-BDNF monoclonal antibody (clone; EPR1292; Abcam, Cambridge, MA, USA) in TBST containing 3% BSA at 4 °C overnight, and then washed three times with TBST. Subsequently, the membranes were incubated at room temperature for 1 h with peroxidase-conjugated secondary antibodies (GE Healthcare Life Sciences) in TBST containing 3% BSA and washed three times with TBST. The signal was detected using Luminata Forte HRP Substrate (Merck Millipore, Billerica, MA, USA). The blot images were captured with Image Quant Las 500 imager (GE Healthcare Life Science), and the exposure time was adjusted such that the intensity of the bands was within the linear range. Quantification of the intensity of BDNF bands was demonstrated using Image Quant TL software (GE Healthcare Life Science). As a control, *E. coli*-derived proBDNF [29] and mBDNF were loaded (Figure 1b and Figure S1). *E. coli*-derived mBDNF was kindly provided by Sumitomo Pharmaceuticals (Osaka, Japan).

For Western blot study (Figure 1c), hippocampal lysates (25 μg) were resolved on 20% SDS-PAGE and then transferred onto PVDF membranes. After that membrane was incubated with a mouse anti-pan-BDNF antibody (clone; 3C11, Icosagen, Estonia). A monoclonal β-actin antibody (clone; AC15, Sigma, USA) was used as a loading control.

### 4.6. Heart Morphology

Heart malformation and intramyocardial hemorrhage were investigated according to a previous report [35]. 

### 4.7. Immunohistochemistry

A purified anti-phospho-TrkB antiserum was generously provided by Professor Moses Chao (Skirball Institute of Biomolecular Medicine, New York University Langone Medical Center, New York, NY, USA). For immunohistochemistry, 10-week-old mice were anesthetized and transcardially perfused with phosphate-buffered saline (PBS) followed by 4% paraformaldehyde in PBS. The brains were postfixed overnight at 4 °C, cryoprotected, and then cut into 30 µm sections. 

Immuno-staining using a rat monoclonal anti-mouse CD31 antibody (1:100, clone MEC 13.3; BD Pharmingen, San Jose, CA, USA) was performed to identify endothelial cells in the mouse heart. Briefly, the sections were treated with 0.1% hydrogen peroxide and then incubated with the primary CD31 antibody. To detect the activated TrkB receptor, the purified anti-phospho-TrkB antiserum was used. Signal amplification was performed using the avidin/biotinylated horseradish peroxidase complex method (Vectastain ABC; Vector Labs, Burlingame, CA, USA). Light microscopy images were obtained using Eclipse E600 Microscope (Nikon, Tokyo, Japan) and a 20× Plan Apo, 0.75 numerical aperture lens objective (Nikon).

### 4.8. Body Weight and Food Intake

The body weights of mice were measured after the tail suspension test. The daily amount of food intake was determined on a per-cage basis from the actual amount of food consumed.

### 4.9. Tail Suspension Test

After 8 weeks of group housing or social isolation, the tail suspension test was performed on an automated tail suspension device (Muromachi Kikai, Tokyo, Japan). According to a previous report [37], animals were suspended from a strain gauge for 6 min. Time spent immobile was recorded in seconds. The settings for the equipment were as follows: time constant = 0.25, gain = 4, threshold 1 = 3, and resolution = 200 ms.

### 4.10. Nest-Building Assessment

Nest-building behavior was assessed after 8 weeks of group housing or social isolation by using a standardized scoring scale described in a previous report [38]. Initially, one piece of nest material (Nestlet, ~3 g; Animec, Japan) was placed on the floor in the center of the home cage (220 × 320 × 135 mm) at 8:00 pm. The nests were assessed 12 h later according to a 5-point scale (see Figure 4a and BOX 1 in the article by Deacon [38]). 

### 4.11. Statistics

All the data are presented as means ± standard errors of the means (SEMs). For statistical analyses, Student’s *t* tests (Figure 2a and Figure S2) and one-way ANOVAs followed by Tukey–Kramer’s multiple-comparison tests (Figure 3 and Figure 4) were used. A *p* value of <0.05 was considered significant.

## Supplementary Materials

Supplementary Materials can be found at https://www.mdpi.com/1422-0067/21/11/3984/s1. Table S1. The frequencies were determined on the basis of genotyping of 63 pups from six independent colonies. Figure S1. Western blotting was performing using hippocampal lysates prepared from 8-week-old BDNF^pro/pro^ mice and 10-day-old BDNF KO^−/−^ and BDNF KO^+/+^ animals. Figure S2. Quantitative real-time PCR analysis. The levels of *Bdnf* mRNA in the hippocampi prepared from BDNF^pro/+^ and BDNF^+/+^ mice (8 weeks old) were measured.

## Abbreviations

BDNF	brain-derived neurotrophic factor
mBDNF	mature form of brain-derived neurotrophic factor
DAPI	4′,6′-diamidino-2-phenylindole dihydrochloride
DG	dentate gyrus
H	hilus
SL	stratum lucidum
