# Supplementary material for: Reduction in BDNF from Inefficient Precursor Conversion Influences Nest Building and Promotes Depressive-Like Behavior in Mice
Source: Int J Mol Sci. 2020 Jun 1;21(11):3984. doi: 10.3390/ijms21113984 (PMC7312902; doi:10.3390/ijms21113984)
Supplement: Supplementary file 1 [file ijms-21-03984-s001.pdf]

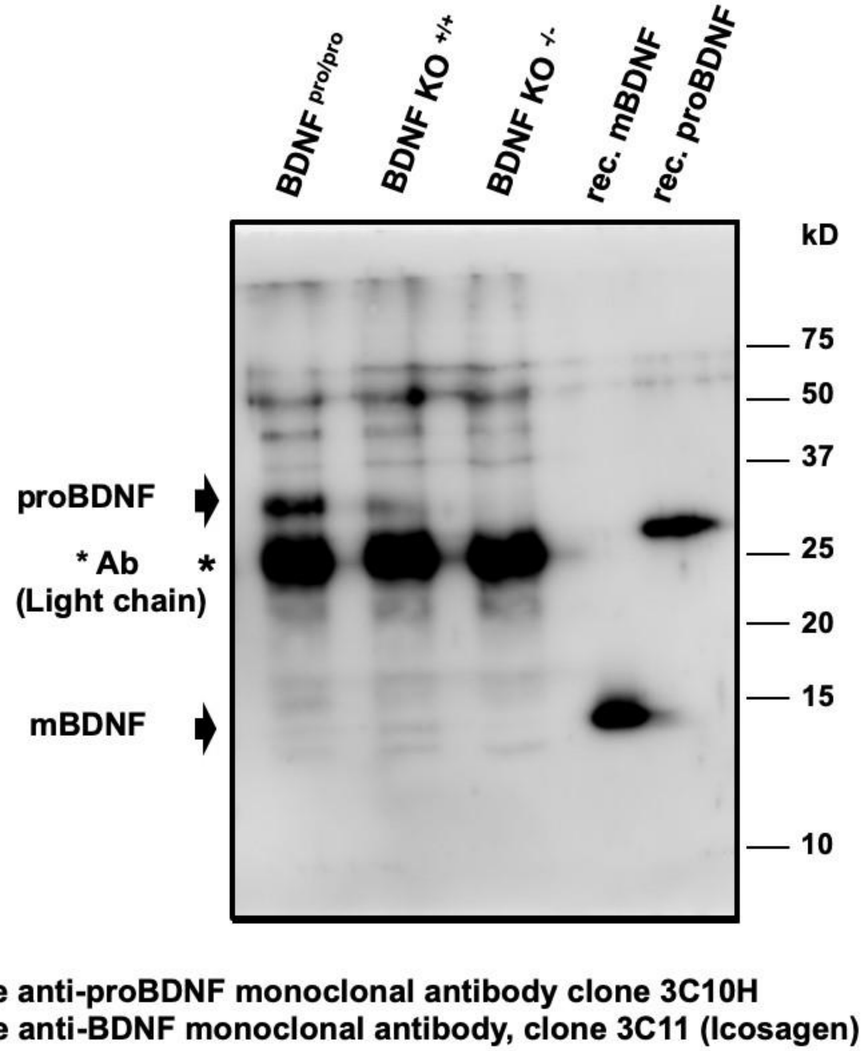

Figure 1. Western blotting was performing using hippocampal lysates prepared from 8-week-old BDNF$^{pro/pro}$ mice and 10-day-old BDNF KO$^{-/-}$ and BDNF KO$^{+/+}$ animals. For immunoprecipitation, hippocampal lysates containing 500 µg protein were incubated with the 3C10H antibody. Next, the immunoprecipitates were electrophoresed on a 20% SDS-polyacrylamide gel. Immunoblotting was performed with a rabbit anti-pan-BDNF monoclonal antibody (EPR1292; Abcam). * indicates light chain of IgG. Note that since the monoclonal antibody recognizing the BDNF pro-domain (3C10H) was used for immunoprecipitation, the proBDNF band was detected but mBDNF was not.

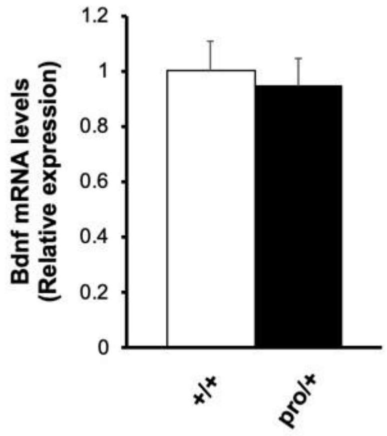

Figure 2. Quantitative real-time PCR analysis. The levels of *Bdnf* mRNA in the hippocampi prepared from $BDNF^{pro/+}$ and $BDNF^{+/+}$ mice (8 weeks old) were measured. All procedures of quantitative real-time PCR analysis are described in the Materials and Methods. Quantitative RT-PCR analyses revealed that there was no significant difference in *Bdnf* expression between the genotypes (n = 3 mice/group). p = 0.30 by t test.

Table 1. Mendelian inheritance frequencies of the animals.

**Parameter**	**BDNF Genotype**		
	**+/+**	**pro/+**	**pro/pro**
No. of pups	16	31	16
Frequency (%)	25.4	49.2	25.4

The frequencies were determined on the basis of genotyping of 63 pups from six independent colonies.
